# Rapid Identification and Susceptibility Testing Directly From Urine Specimens, Reducing Turnaround Time, a Step Towards Advancing Diagnostic Stewardship

**DOI:** 10.7759/cureus.93441

**Published:** 2025-09-28

**Authors:** Maitrayee Narayan, Sarita Mohapatra, Bimal Das, Kalaivani Mani, Hitender Gautam, Seema Sood, Benu Dhawan

**Affiliations:** 1 Department of Microbiology, All India Institute of Medical Sciences, New Delhi, IND; 2 Department of Biostatistics, All India Institute of Medical Sciences, New Delhi, IND

**Keywords:** diagnostic stewardship, direct antimicrobial susceptibility testing, direct identification, turnaround time, urinary tract infection

## Abstract

Background

Urinary tract infections (UTIs) are the most common bacterial infections with a turnaround time (TAT) of 48-72 hours for routine culture and antimicrobial susceptibility testing (AST). Here, we aimed to improve diagnostic stewardship by reducing TAT for the most commonly encountered uropathogens, that is, *Escherichia coli *and *Klebsiella pneumoniae* by combining the usage of matrix-assisted laser desorption/ionization time-of-flight mass spectrometry (MALDI-TOF MS) for the direct identification of pathogens from urine specimen, along with direct AST at different time points.

Methods

Urine specimens were examined by compound microscopy under high power field (HPF). Thirty-six urine specimens and 25 urine specimens were included for the standardization phases of direct MALDI-TOF MS and direct AST, respectively, while 65 and 55 specimens were included for the validation phases, respectively. AST was read at four hours, six hours, eight hours and 16-18/16-20 hours using the Clinical and Laboratory Standards Institute (CLSI) M100, 34th edition, 2024 breakpoints for direct blood cultures and the European Committee on Antimicrobial Susceptibility Testing (EUCAST) version 7.1, 2024 breakpoints for short incubation disc diffusion. Culture-based MALDI-TOF MS identification, culture-based VITEK II (BioMérieux, Marcy-l'Étoile, France) or AST were considered the reference standard. All readings were compared with reference standard values for statistical and agreement analysis.

Results

Using direct MALDI-TOF MS, 88.2% (30/34) and 90.7% (59/65) of uropathogens were correctly identified in the standardization and validation phases, respectively. The results of direct AST showed that most antibiotics had a categorical agreement of greater than 90%, and acceptable error rates by six to eight hours, resulting in a TAT reduction in the range of 39-43 hours.

Conclusion

This proof-of-concept study using a combined protocol is a feasible way to give final identification and AST results within six to eight hours of sample reception, thus providing an impetus to diagnostic stewardship and ensuring timely intervention.

## Introduction

Antimicrobial resistance (AMR) is an ongoing worldwide epidemic fueled by the inappropriate usage of antibiotics. Urinary tract infections (UTIs) are one of the most common conditions treated with empirical antibiotics, thus contributing predominantly to the burden of AMR. The main causative agents of UTIs in both hospitalized and community settings are *Escherichia coli* (64%-75%), and *Klebsiella pneumoniae* (6%-19%) [1-4].

Urine culture and antimicrobial susceptibility testing (AST) remain the most frequently ordered tests and the cornerstone for the diagnosis and management of UTIs in healthcare settings. Given that the turnaround time (TAT) for conventional culture and AST is approximately 48-72 hours, empirical treatment is initiated for a majority of the patients. In the Indian context, percentage resistance of *E. coli* and *K. pneumoniae* against common oral antibiotics used in UTI, that is, fluoroquinolones, third and fourth generation cephalosporins, and cotrimoxazole, is above 50%. While urinary isolates of *E. coli *exhibit less than 10% resistance to nitrofurantoin and fosfomycin, *K. pneumoniae* currently exhibits more than 50% resistance to nitrofurantoin [5]. Therefore, diagnostic stewardship (DxS) is crucial because it encourages the best urine culture and reporting procedures, enabling prompt intervention and avoiding the overuse of antibiotics. In keeping with the analytical component of DxS, a rapid technique alternative to conventional culture and AST is much needed to ensure targeted treatment as early as possible.

In this proof-of-concept study, we aimed to reduce the TAT for identification and susceptibility testing of the most commonly found uropathogens *E. coli* and *K. pneumoniae*, directly from urine samples using a novel combination of matrix-assisted laser desorption/ionization time-of-flight mass spectrometry (MALDI-TOF MS) and direct AST using disc diffusion at different time points. The application of rapid/direct AST breakpoints has till now been defined only for blood cultures. In this study, we attempted to determine the feasibility of achieving reliable results within four to eight hours using these rapid breakpoints for direct AST using urine specimens.

## Materials and methods

The study was carried out for a duration of six months (April to September 2024) at the bacteriology laboratory of the All India Institute of Medical Sciences, New Delhi. Ethical permission was obtained from the Institute Ethics Committee.

Urine specimens received at our laboratory for culture and AST within two hours of sample collection from patients with a clinical diagnosis of UTI were screened for this study using direct microscopy. The study was conducted in two stages: (a) direct identification by MALDI-TOF MS (Direct MALDI-TOF MS) and (b) direct AST.

**Figure 1:**
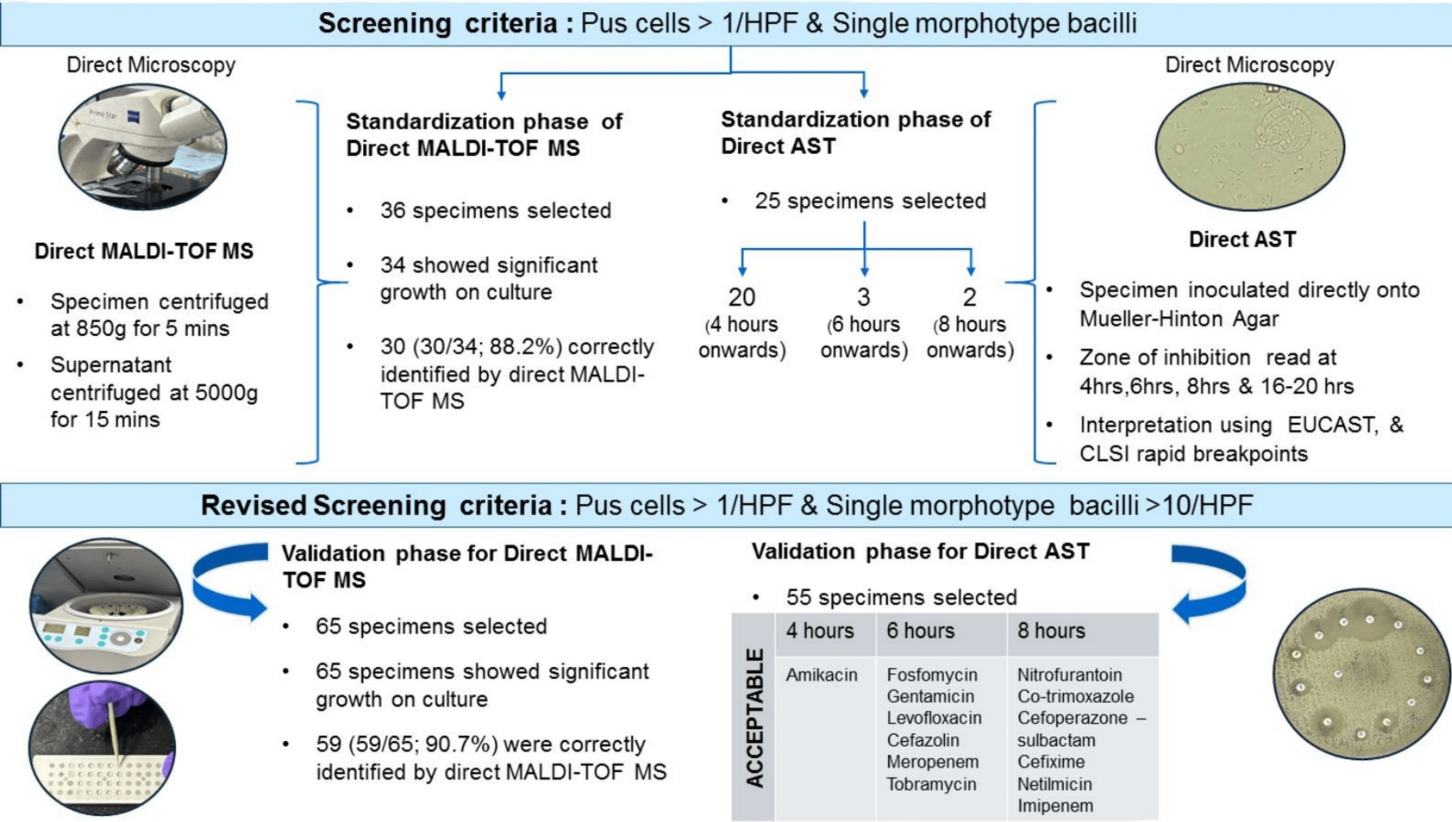
Diagram showing workflow using combined protocol of Direct MALDI-TOF MS and Direct AST The first part of the diagram shows the standardization phase of direct identification by MALDI-TOF MS and direct AST. No zone diameters were read for direct AST during the standardization phase. The second part of the diagram shows the validation phase using direct MALDI-TOF MS and direct AST using a modified screening criteria. The results of direct AST for individual antibiotics from the time point onwards, where all components of agreement analysis are within acceptable limits, have been shown as acceptable. MALDI-TOF MS: Matrix-assisted laser desorption ionization-time of flight mass spectrometry; AST: antimicrobial susceptibility testing.

The workflow of the combined protocol of direct MALDI-TOF MS and direct AST is shown in Figure 1. Since this is a proof-of-concept study, a minimum sample size of 25-30 was required. Therefore, 36 and 65 specimens were included in the standardization and validation phases of direct identification by MALDI-TOF MS, respectively. For the standardization and validation phases of direct AST, 25 specimens and 55 specimens were included, respectively.

Direct identification by MALDI-TOF MS

Direct microscopy was performed under high power field (HPF) for the urine specimens. The inclusion criteria used was: urine samples with ≥one leucocyte per seven HPF (≥10^4^ leucocytes/ml) along with the presence of single morphotype bacilli. The exclusion criteria comprised: urine samples showing the absence of pyuria, presence of cocci and/or bacilli of different morphotypes, presence epithelial cells and/or budding yeast cells [6]. Thirty-six specimens that fulfilled the inclusion criteria from among 590 specimens were selected for further processing in the standardization phase.

Direct identification of bacteria from the urine specimen was done by MALDI-TOF MS (Vitek-MS system, BioMérieux, Marcy-l'Étoile, France) using an in-house modification of the protocol described by Zboromyrska et al. [7].

The selected urine specimens were divided into aliquots of 7 to 10 ml for centrifugation. The remaining volume of specimens were stored at 4°C for the next part of the study, which was carried out within a few hours of the first. The specimens were centrifuged at 850g for five minutes. The supernatant was transferred to another tube ensuring no contamination by the pellet, which consisted of cellular debris. The supernatant was then again centrifuged at 5000g for 18 minutes. Following the second centrifugation, the supernatant was discarded and the newly formed pellet consisting of bacterial cells was washed with de-ionized water twice. The pellet was then dissolved in 3 µl of de-ionized water and pipetted directly onto the MALDI plate. Before fully drying, the MALDI target was overlaid with 1 µl of matrix solution (saturated solution of α-cyano-4-hydroxy cinnamic acid) to allow proper cross-linking between the sample and matrix, following which it was air-dried.

In parallel, the urine samples were also plated on cysteine lactose electrolyte deficient agar (CLED) media and incubated overnight for any significant growth. Culture-based MALDI-TOF MS was considered the reference standard for pathogen identification, and the results of direct identification were compared with it. A colony count of 10^5 ^colony forming units (CFU)/ml was considered significant. In addition, if pus cells were found to be >20/HPF, the initial centrifugation step at 850g was repeated twice with the supernatant before proceeding further.

For the validation phase, the inclusion criteria used was: urine samples with ≥one leucocyte per seven HPF (≥10^4^ leucocytes /ml) and single morphotype bacilli >10/ HPF (>7x10^5^ CFU/ml). The exclusion criteria remained the same as the standardization phase. This modification of the inclusion criteria was based on the results of direct AST standardization. Using the modified criteria, 65 urine specimens were selected from among 930 specimens. Since *E.coli* and *K. pneumoniae* constitute greater than 80%-90% of hospital-acquired and community-acquired UTI, we targeted only those urine specimens which grew monobacterial colonies of either *E.coli *or *K. pneumoniae * with significant colony counts (≥10^5^ CFU/ml).

Direct AST 

The standardization phase for direct AST was done to evaluate the screening criteria, which would allow visible growth on the Mueller-Hinton Agar (MHA) plate at the earliest time point of four hours. Out of the 36 urine specimens screened for direct MALDI, 25 with sufficient remaining volume were selected for the standardization of direct AST. These urine specimens were inoculated onto MHA. Seven to 10 ml of urine was poured onto a 150 mm plate and rotated around the plate till all areas were uniformly covered. The excess urine was then decanted. Moisture from the plates was allowed to evaporate and antimicrobial discs used for routine AST were prepared as per our standard operating procedure (SOP). The plates were then inverted and incubated at 37°C. The initial bacterial load in the specimen had to be sufficient to allow the formation of zone diameters at four hours onwards. Among the 25 urine specimens selected for standardization of direct AST, the zone of inhibition appeared at four hours in 80% (20/25) of specimens, in which >10 bacilli/HPF had been noted during screening. For three specimens, zones of inhibition appeared at six hours and for the remaining two after eight hours. All five specimens had shown <10 bacilli/HPF during screening. Therefore, on the basis of results observed for the standardization phase of direct AST, a modified inclusion criteria including a direct microscopy cut-off of monomorphic bacterial counts ≥10/HPF was taken for the validation of both stages of the study. Zone diameters for direct AST were not read for the standardization phase.

Quality control (QC) was performed weekly as per standard disc diffusion QC procedures for *E. coli *ATCC 25922. QC for MHA was performed by testing trimethoprim-sulfamethoxazole disks using *Enterococcus faecalis* ATCC 29212 [8].

For the validation of the direct AST, 55 specimens were selected as per the modified inclusion criteria, from among the specimens screened for the validation phase of direct MALDI. The results of the direct AST were read at four, six, eight and 16-20 hours following the rapid antimicrobial susceptibility testing (RAST) European Committee on Antimicrobial Susceptibility Testing (EUCAST) guidelines, and at eight and 16-18 hours following the Clinical and Laboratory Standards Institute (CLSI) guidelines for direct blood cultures [8,9]. Drugs interpreted as per EUCAST breakpoints included amikacin (30 µg), gentamicin(10 µg), tobramycin(10 µg), ciprofloxacin (5 µg), levofloxacin (5 µg), cotrimoxazole (1.25/23.75 µg), imipenem (10 µg) and meropenem (10 µg). Interpretation as per the CLSI breakpoints for direct blood cultures included ciprofloxacin (5 µg), cotrimoxazole (1.25/23.75 µg), meropenem (10 µg), tobramycin (10 µg), and ceftazidime (30 µg). Zone diameters for cefotaxime (30 µg) were interpreted as per those of ceftriaxone given for direct blood cultures (CLSI). For netilmicin (30 µg), cefoperazone-sulbactam (75/30 µg), nitrofurantoin (300 µg), fosfomycin (200 µg), cefepime (30 µg), cefixime (5 µg), cefuroxime (30 µg) and cefazolin (30 µg), no rapid/direct breakpoints are available either in CLSI or EUCAST. For these drugs, all four timepoints were interpreted using CLSI conventional disc diffusion breakpoints. As piperacillin-tazobactam was not available to us in the disc concentration prescribed for RAST EUCAST, it could not be tested.

Culture followed by the interpretation of VITEK II (BioMérieux, Marcy-l'Étoile, France), the minimum inhibitory concentration (MIC) values using CLSI was considered the reference standard for AST. Antibiotics not in the VITEK II panel included cefazolin, cefixime, cefotaxime, ceftazidime, netilmicin, levofloxacin, tobramycin, and nitrofurantoin. For these antibiotics, culture followed by the results of disc diffusion at 16-20 hours was considered the reference standard, with zone diameters being interpreted as per CLSI conventional disc diffusion breakpoints for AST.

Concordance between the results of the direct AST and the reference standard was calculated using agreement analysis with acceptable criteria for categorical agreement (CA) being greater than 90%, minor error (MnE) being less than 10 %, major (ME) and very major errors (VME) being less than 3% respectively [10]. Categorical agreement is the percentage of results (susceptible, intermediate or resistant) that match the gold standard. Minor errors are results showing intermediate susceptibility by the test method, which are either susceptible or resistant by the gold standard and vice-versa, calculated as a fraction of the total number of isolates. Major errors denote isolates that are resistant going by the test method but susceptible by gold standard and are calculated as a fraction of all susceptible isolates as per the gold standard. Very major errors denote isolates, which are susceptible by the test method but resistant as per the gold standard and are calculated as a fraction of all resistant isolates as per the gold standard. For drugs not in the VITEK II panel, zone diameters at different time points of direct AST were also compared with the reference standard zone diameters using Bland Altman plots, which depict the agreement between two measurement techniques against their average.

The TAT for each specimen was taken as the first time point where the susceptibility result of the rapid AST matched the result of the reference standard for that specimen. A mean TAT was calculated from the TAT of each of the 55 specimens tested. The reduction in TAT (95% CI) was calculated as the difference in time (hours) between the results of conventional reporting and those as per the combined protocol.

## Results

Thirty-six specimens were screened for direct identification using MALDI-TOF MS, of which 34 (34/36, 94%) showed significant growth on culture (gram-negative bacilli (GNB): 33 (33/36, 91.6%); gram-positive cocci (GPC): 1 (1/36, 2.7%)) (Table 1).

**Table 1:** Results of the standardization of Direct MALDI-TOF MS MALDI-TOF MS: Matrix-assisted laser desorption/ionization time-of-flight mass spectrometry.

Organisms (No. of isolates)	Identification by culture (% isolates)	Identification by direct MALDI-TOF MS (% isolates)
*Escherichia coli* (15 )	15 (41.6)	13 (36.1)
Klebsiella pneumoniae (14)	14 (38.8)	14 (38.8)
*Pseudomonas aeruginosa* (3)	3 (8.3)	2 (5.5)
*Providencia rettgeri* (1)	1 (2.7)	1 (2.7)
*Enterococcus faecium* (1)	1 (2.7)	Not detected
More than 3 types of GNB colonies (1)	Not processed further	Not detected
Insignificant GPC colonies (1)	Not processed further	Not detected

Among the 34 GNB isolates, with significant growth on culture, 30 (30/34, 88.2%) were correctly identified by the direct method. The performance characteristics of direct MALDI-TOF MS showed a sensitivity of 88%, specificity of 100%, positive predictive value of 100% and a negative predictive value of 33%.

In the validation phase, a total of 65 urine specimens were tested for direct identification, and all showed monobacterial colonies with significant colony counts on culture. Fifty-nine specimens were correctly identified by direct MALDI-TOF MS, showing a concordance of 90.7% with the gold standard (36 (61.01%): *E.coli* and 19 (32.20%): *K. pneumoniae*). The remaining four specimens were identified as *P. aeruginosa* and excluded from the study.

In the validation phase, amikacin was the only antibiotic, where all parameters of agreement analysis were within an acceptable range at four hours. Fosfomycin, gentamicin, levofloxacin, cefazolin, meropenem, and tobramycin showed all parameters of agreement analysis to be within an acceptable range from six hours onwards, from eight hours onwards for nitrofurantoin, co-trimoxazole, cefixime, cefoperazone-sulbactam, netilmicin and imipenem and by 16-20 hours for cefuroxime (Table 2).

**Table 2:** Rates of very major error (VME), major error (ME) and minor error (MnE) at different time points ^*^Interpretation at different time points with reference to CLSI breakpoints for direct AST. ^**^Interpretation at different time points with reference to CLSI  breakpoints for culture-based AST. ^a^Categorical agreement and error rates within acceptable range from 4 hours. ^b^Categorical agreement and error rates within acceptable range from 6 hours. ^c^Categorical agreement and error rates within acceptable range from 8 hours. ^d^Categorical agreement and error rates within acceptable range from 16-20 hours. EUCAST: European Committee on Antimicrobial Susceptibility Testing; CLSI: Clinical and Laboratory Standards Institute.

Breakpoints used for Interpretation of direct AST from urine samples												
Rapid Antimicrobial Susceptibility breakpoints (EUCAST)												
Anti-microbial agent	4 hours			6 hours			8 hours			16-20 hours (EUCAST)/16-18 hours (CLSI)		
	MnE (%)	ME (%)	VME (%)	MnE (%)	ME (%)	VME (%)	MnE (%)	ME (%)	VME (%)	MnE (%)	ME (%)	VME (%)
Amikacin^a^	7.2	2.5	0	1.8	2.5	0	1.8	0	0	1.8	0	0
													Gentamicin^b^	5.4	11.7	0	1.8	2.9	0	1.8	0	0	1.8	0	0
Tobramycin^b^	3.6	15	0	0	3	0	0	0	0	0	0	0													
													Ciprofloxacin	3.6	14.2	8.8	7.2	0	13.3	5.4	0	15.5	5.4	0	15.5
Levofloxacin^b^	9	17.6	0	7.2	0	0	3.6	0	0	5.4	0	0													
													Imipenem^c^	12.7	13.5	0	12.7	2.7	0	3.6	0	0	1.8	0	0
Meropenem^b^	3.6	18.9	5.5	5.4	2.7	0	1.8	0	0	1.8	0	0													
													Cotrimoxazole^c^	0	36.8	0	0	10.5	2.7	0	0	2.7	0	0	2.7
Direct CLSI^* ^or Conventional CLSI breakpoints^**^																									
Netilmicin^**c^	3.6	23	0	1.8	5.1	0	1.8	0	0	1.8	0	0
Tobramycin*^c^	-	-	-	-	-	-	0	0	0	0	0	0
													Ciprofloxacin*	-	-	-	-	-	-	10.9	0	8.8	10.9	0	8.8
Ceftazidime*	-	-	-	-	-	-	9	8.6	0	7.2	4.3	0													
Cefotaxime*	-	-	-	-	-	-	5.4	23.8	0	5.4	23.8	0
													Cefoperazone Sulbactam**^c^	27.2	24	0	12.7	6	0	5.4	0	0	0	0	0
Fosfomycin**^b^	5.5	19.3	0	5.5	0	0	0	0	0	0	0	0
Nitrofurantoin**^c^	12.7	20.4	0	9	9	0	5.4	2.2	0	3.6	0	0
Cefazolin**^b^	0	8.3	0	0	0	0	0	0	0	0	0	0
Cefixime**^c^	3.6	33.3	0	1.8	16.6	0	1.8	0	0	1.8	0	0
Cefepime**	30.9	24	0	16.3	12	0	5.4	4	0	5.4	4	0
Cefuroxime**^d^	12.7	10	0	10.9	10	0	10.9	0	0	9	0	0
Meropenem*^c^	-	-	-	-	-	-	1.8	0	0	1.8	0	0
Cotrimoxazole*^d^	-	-	-	-	-	-	-	-	-	0	0	2.8

**Figure 2:**
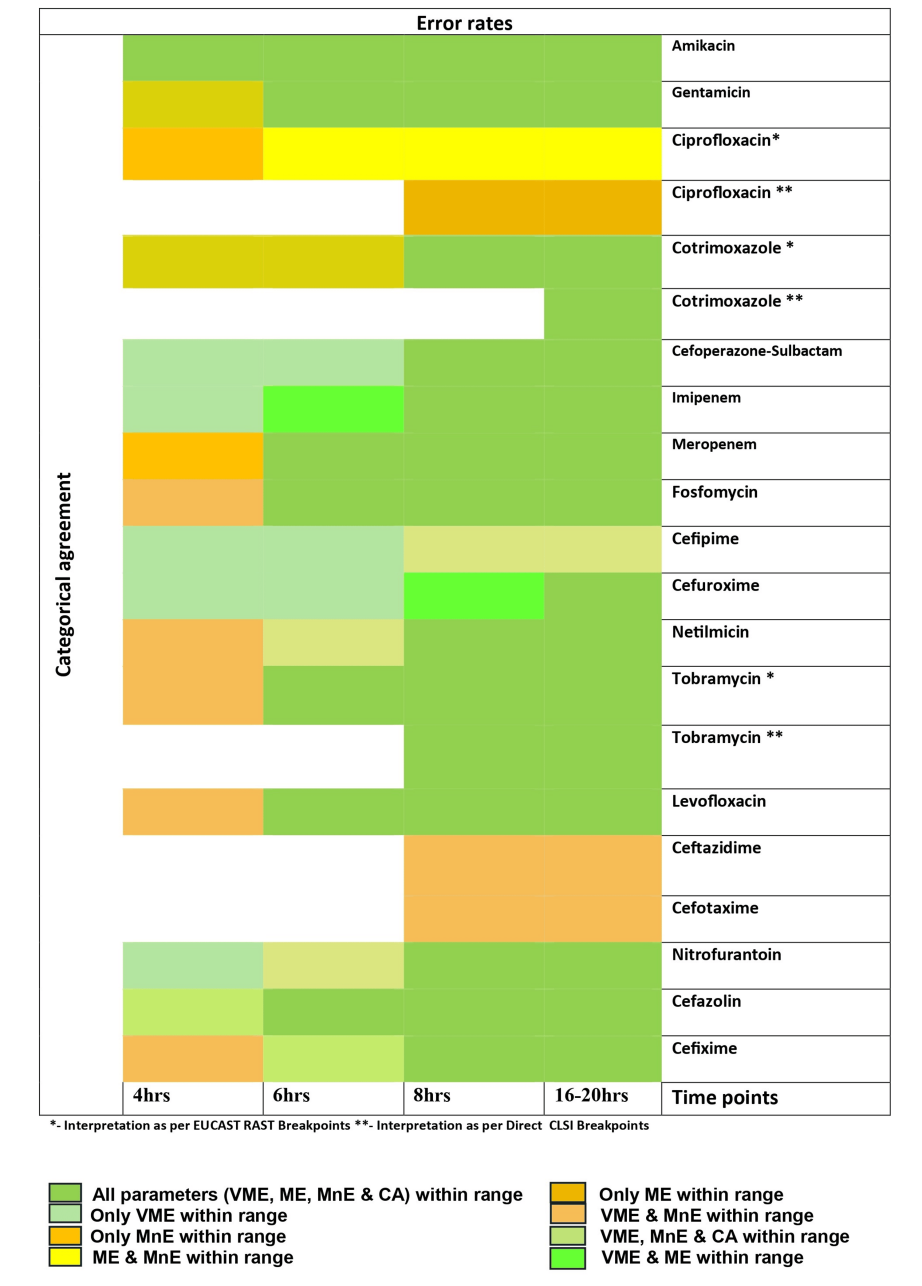
Heat map showing acceptable time points for interpretation of direct AST based on error rates and categorical agreement Image shows components of agreement analysis (minor error (MnE), major error (ME), very major error (VME) and/or categorical agreement (CA) which are within acceptable limits at different time points for each antibiotic. Heat map generated using Microsoft Word (Office 365, Microsoft, Redmond, WA). Image credit: Dr. Maitrayee Narayan.

However, VME rates were zero by four hours for all antibiotics except ciprofloxacin and meropenem. For ceftazidime and cefotaxime, zone diameters were interpreted only at eight hours and 16-18 hours, showing VME to be zero and MnE to be within an acceptable range for both time points. For cefepime, VME rates were zero at all time points with CA and MnE within an acceptable range by eight hours. However, for ciprofloxacin, majority of the parameters (VME, MnE and CA) were out of range at all time points. We have attempted to show acceptable time points for the interpretation of direct AST for our study based on categorical agreement and error rates in a heat map (Figure 2).

**Figure 3:**
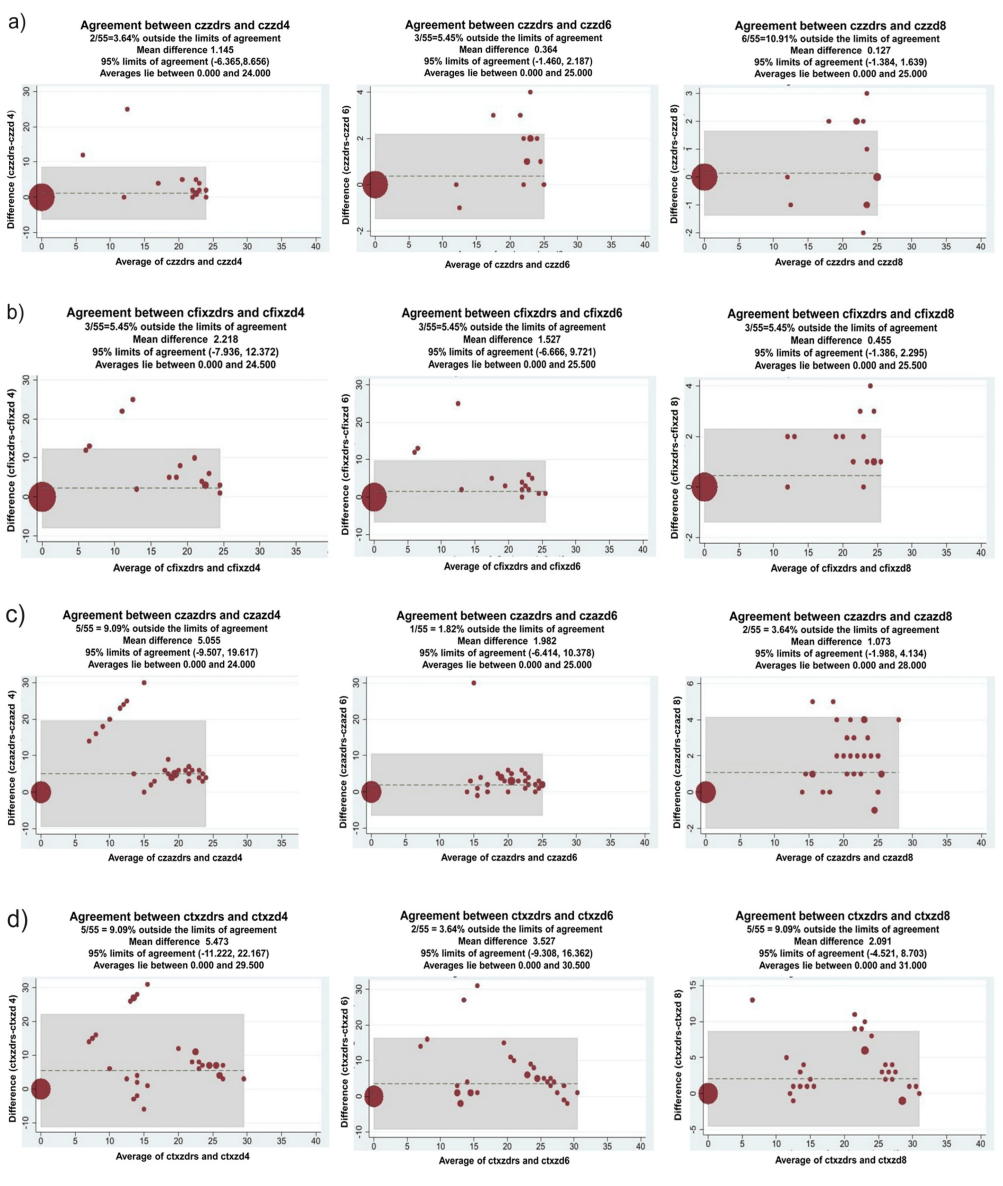
Correlation between zone diameters of direct antimicrobial susceptibility testing (AST) and conventional AST using Bland Altman plot Y-axis: Difference between the reference standard zone diameter and direct AST zone diameter; X-axis: Average of the reference standard zone diameter and direct AST zone diameter; Shaded areas represent 95% limits of agreement between the direct AST and conventional AST. (a) czzdrs: Cefazolin zone diameter reference  standard, czzd4: cefazolin zone diameter (4 hours), czzd6: cefazolin zone diameter(6 hours), czzd8: cefazolin zone diameter (8 hours). (b) cfixzdrs: Cefixime zone diameter reference standard, cfixzd4: cefixime zone diameter (4 hours), cfixzd6: cefixime zone diameter (6 hours), cfixzd8: cefixime zone diameter(8 hours). (c) czazdrs: Ceftazidime zone diameter reference standard, czazd4: ceftazidime zone diameter (4 hours), czazd6: ceftazidime zone diameter (6 hours), czazd8: ceftazidime zone diameter (8 hours). (d) ctxzdrs: Cefotaxime zone diameter reference standard, ctxzd4: cefotaxime zone diameter (4 hours), ctxzd6: cefotaxime zone diameter (6 hours), ctxzd8: cefotaxime zone diameter (8 hours).

**Figure 4:**
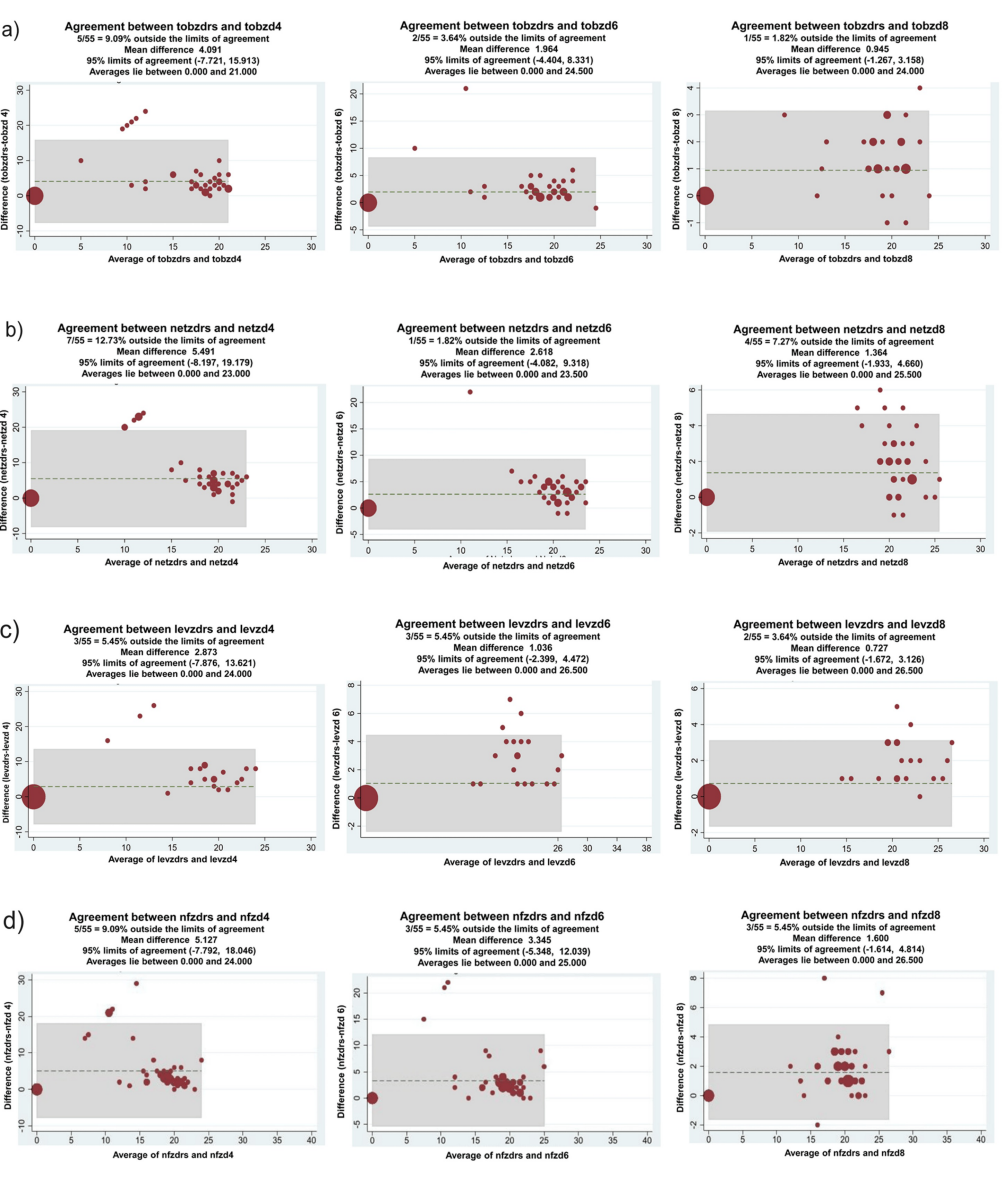
Correlation between zone diameters of direct antimicrobial susceptibility testing (AST) and conventional AST using Bland Altman plot (a) tobzdrs: Tobramycin zone diameter reference standard, tobzd4: tobramycin zone diameter (4 hours), tobzd6: tobramycin zone diameter (6 hours), tobzd8: tobramycin zone diameter (8 hours). (b) netzdrs: Netilmicin zone diameter reference standard, netzd4: netilmicin zone diameter (4 hours), netzd6: netilmicin zone diameter (6 hours), netzd8: netilmicin zone diameter (8 hours). (c) levzdrs: Levofloxacin zone diameter reference standard; levzd4: levofloxacin zone diameter (4 hours); levzd6: levofloxacin zone diameter (6 hours); levzd8: levofloxacin zone diameter (8 hours). (d) nfzdrs: Nitrofurantoin zone diameter reference standard; nfzd4: nitrofurantoin zone diameter (4 hours); nfzd6: nitrofurantoin zone diameter (6 hours); nfzd8: nitrofurantoin zone diameter (8 hours).

For those antimicrobials that were not in the VITEK II panel, disk diffusion zones at different time points were compared with the reference standard values using Bland Altman plots (Figures 3, 4).

Zone diameters at all time points showed excellent correlation, with most showing a correlation of >90% from four hours onwards. The exception to this was netilmicin (87% correlation at 4 hours).

The mean reduction in TAT using direct AST was in the range of 35-43 hours for different antimicrobials compared to conventional processing time (Table 3).

**Table 3:** Mean reduction in turnaround time (TAT) among all urine isolates for direct antimicrobial susceptibility testing

Antimicrobial	Mean reduction of TAT (in hours)±Margin of error at 95% CI
Amikacin	43.01±1.57 (±3.65%)
Netilmicin	42.76±1.57 (±3.68%)
Gentamicin	42.76±1.63 (±3.83%)
Tobramycin	43.70±0.21 (±0.49%)
Ciprofloxacin	35.96±4.52 (±12.57%)
Levofloxacin	42.8±1.57 (±3.69%)
Cotrimoxazole	43.63±0.25 (±0.58%)
Fosfomycin	43.63±0.25 (±0.58%)
Nitrofurantoin	41.27±2.26 (±5.49%)
Cefazolin	43.96±0.07 (±0.16%)
Cefixime	42.94±1.57 (±3.67%)
Cefuroxime	39.6±3.38 (±8.54%)
Cefepime	40.54±2.62 (±6.48%)
Cefoperazone sulbactam	41.92±1.63 (±3.90%)
Meropenem	43.52±0.28 (±0.66%)
Imipenem	42.98±0.74 (±1.74%)

Details of categorical agreement for all isolates and error rates for each drug/organism combination are provided in the Appendices (Tables 4 to 9).

## Discussion

Diagnostic stewardship is based on ordering the right test for the right patient at the right time. Reducing TAT for diagnostic tests using rapid techniques is a key component of DxS. As UTI syndromes are one of the most commonly encountered infectious diseases in clinical practice, the rationale for this study is based on the need for a reduction in TAT of urine culture and AST.

Modern diagnostic techniques for UTI with reduced TAT include molecular techniques like polymerase chain reaction, MALDI-TOF MS and forward light scattering [11]. The above methods are qualitative, and this is a major disadvantage as the diagnosis of UTI rests on a significant colony count. In addition, these molecular techniques are expensive, require extensive initial processing and may not provide a result for antimicrobial susceptibility testing as detection of resistance genes may not translate to resistance in-vivo. However, studies done on the direct identification of pathogens using MALDI-TOF MS have shown a concordance ranging from 88% to 94% [7,12-14].

Zboromyrska et al., using flowcytometry to ascertain bacterial load, subjected samples with ≥5000 bacteria/µl to identification via direct MALDI-TOF MS [7]. Wei Li et al. processed the bacterial pellet with 70% formic acid, followed by the addition of acetonitrile, to increase the yield of direct identification [12]. Ferreira et al. used a modified ethanol formic acid extraction procedure for better yield. Other techniques aimed at increasing the yield include using pre-treatment of urine with sodium dodecyl sulfate (SDS) to lyse the cell and a fast lipid analysis technique (FLAT) [14,15].

In our study, we used a modification of the protocol as per Zboromyrska et al. [7] and have achieved a greater than 90% concordance of direct identification by MALDI-TOF MS with culture, which is comparable to the above protocols. In addition, we have screened samples for direct identification using urine wet mount examination. During the standardization phase of direct AST, counts of bacilli >10/HPF in wet mount examination were shown to allow visible bacterial growth after four hours of incubation. Thus, as the inoculating volume had been around 10 ml of urine, this would correspond to a bacterial count >10^5 ^CFU/ml, which is comparable to the above protocols.

Earlier studies on direct AST focused on using the VITEK-II system by preparing a 0.5 McF solution using the bacterial pellet from a pre-processed urine specimen or directly from the urine specimen itself [16,17]. Both studies showed an overall decrease in TAT with the former having a TAT of six to eight hours for *Enterobacterales* specifically. Our study compares favourably with these and allows reporting of AST for most drugs by six hours in samples with a similar bacterial count, as the above studies.

Breakpoints for the performance of disk diffusion directly from positive blood cultures at eight to 10 hours and 16-20 hours are elucidated in CLSI [8]. Similarly, EUCAST provides instructions for rapid antimicrobial susceptibility testing (RAST) from positive blood culture bottles, to be read at four, six, eight and 16-18 hours [9]. In a study done on direct AST, EUCAST RAST-based interpretation was performed at eight hours for clinical urine samples and compared with conventional ASTs [18]. To the best of our knowledge, no other study till now has extrapolated RAST to urine samples at eight hours and none have extrapolated RAST to urine samples at four and six hours or as per CLSI to samples at eight and 16-20 hours. Mugiraneza et al. found a categorical agreement of RAST with conventional AST for urine specimens at eight hours was 93.3% showing 4.9% minor error, 0.6% major error and 1.2% very major error [18]. Our study corresponds well with this finding, having a cumulative categorical agreement of 95.4% at eight hours, with 3.5% minor error, 0.5% major error and 1.9% very major error. 

Having used breakpoints as provided for rapid/direct positive blood cultures, we found that the comparison of disc diffusion reading at different time points with reference standard values showed the best interpretative breakpoints within eight hours. Except ciprofloxacin, all antibiotics showed an acceptable VME by four to eight hours. The mean reduction in TAT for most antimicrobials was in the range of 40-43 hours, whereas for cefuroxime, it was in the range of 39 hours (Table 3). This reduction in TAT would be of great value in furthering the role of DxS by shifting the focus from empirical prescriptions to targeted therapy and antibiotic de-escalation. As per the Infectious Diseases Society of America (IDSA) 2010 clinical practice guidelines, empirical treatment may be given in uncomplicated cystitis, acute pyelonephritis and in asymptomatic bacteriuria, where further processing is recommended in pregnant females and in those undergoing endourological procedures [4,19]. The downside of empirical broad-spectrum antibiotic therapy is collateral damage or the rise in antimicrobial resistance. Acute uncomplicated cystitis, being the most common indication for antimicrobial exposure in an otherwise healthy population, is the most important contributor to collateral damage [4]. On the other hand, complications are the issue in acute pyelonephritis, causing an overall mortality ranging from 10%-20%, which may be even higher in special population groups such as pregnant females, elderly population, diabetics, and those with long-term indwelling catheters. The use of direct identification and rapid antimicrobial susceptibility testing can reduce collateral damage, emergence of drug resistance and might allow timely targeted intervention with better outcomes.

In addition, the initial screening of urine specimens allowed for reflex urine culture in this study, which in itself is an important component of DxS [20]. For most cases, interpretation via RAST EUCAST parameters provided a better concordance compared to CLSI. CLSI breakpoints, instead of EUCAST, were used for the interpretation of reference standard results and for the time-point interpretation of antibiotics without rapid/direct zone diameters, as this enabled the categorization for intermediate zones, which are better defined in CLSI compared to EUCAST. Although the area of technical uncertainty (ATU) as per EUCAST has a different explanation compared to the intermediate category (I) as per CLSI, the ATU category forms a continuum between the susceptible and resistant categories in RAST and thus isolates falling in this category were taken as intermediate values for the calculation of error rates.

The limitations of this study are that the sample size of our study is small. Variability in sample quality, differences in laboratory processing techniques, representativeness of the selected site or possible biases in sampling locations have not been studied. Therefore, the generalizability of findings cannot be commented upon. However, this is a proof-of-concept study and further studies using a larger number of isolates with multi-center validation would be required to adequately judge the merits of the urine direct AST and the inconsistencies seen for ciprofloxacin. In addition, we did not pointedly screen urine specimens for the presence of antimicrobials. However, this being at best a feasibility study, the use of a colony count of ≥10^5 ^CFU/ml for the reference standard precludes the presence of antimicrobials in the specimen to a degree, which might affect parameters of susceptibility. Another limitation is that urine screening by microscopy is subjective and cannot at times reliably differentiate a monomorphic bacterial population from a polymorphic one, causing an erroneous result in the reading of the direct AST plate. Besides, the microscopy screening criteria required bacterial counts to be greater than or equal to 10^5^/ml, which makes this combined protocol ineffective for lower bacterial counts in the urine sample. Moreover, this study focused only on *E. coli* and *K. pneumoniae* and did not include other *Enterobacterales* or non-*Enterobacterales*, commonly seen in hospital-acquired infections.

## Conclusions

The combined protocol for direct identification of the uropathogen from samples using MALDI-TOF MS and direct AST is a promising method of reducing the TAT by 39-43 hours. With the exception of a few drugs, such as ciprofloxacin, this proof-of-concept study showed good concordance of the combined protocol with the reference standard. The use of this protocol would allow provisional identification and AST results within six to eight hours of sample reception. This would be helpful in starting prompt and targeted therapy for UTIs, helping to advance antimicrobial stewardship and decreasing AMR in the near future. However, further studies with larger sample sizes and multi-center validation would be required to assess the generalizability of these results.

## Appendices

**Table 4:** Categorical agreement of direct AST (total no. of isolates=55) using rapid antimicrobial susceptibility testing (RAST) breakpoints as per EUCAST Reference Standard was either conventional antimicrobial susceptibility testing (AST) or VITEK following 16-18 hours of culture. Abbreviations: RAST (EUCAST): Rapid Antimicrobial Susceptibility Testing (as per the European Committee on Antimicrobial Susceptibility Testing); CA: categorical agreement; S: susceptible; ATU: area of technical uncertainty; R: resistant.

Anti-microbial agent	4 hours				6 hours				8 hours				16 hours				Reference Standard		
Interpretation as per RAST (EUCAST)	S	ATU	R	CA (%)	S	ATU	R	CA (%)	S	ATU	R	CA (%)	S	ATU	R	CA (%)	S	ATU	R
Amikacin	35	3	17	90.9	38	0	17	96.3	40	0	15	98.1	40	0	15	98.1	39	1	15
Gentamicin	28	3	24	87.2	33	1	21	96.3	34	1	20	98.1	34	1	20	98.1	34	0	21
Ciprofloxacin	12	2	41	81.8	15	1	39	81.8	17	0	38	81.8	17	0	38	81.8	7	3	45
Cotrimoxazole	12	0	43	87.2	18	0	37	94.5	20	0	35	98.1	20	0	35	98.1	19	0	36
Imipenem	28	6	21	78.1	32	6	17	85.4	35	3	17	96.3	36	2	17	98.1	37	0	18
Meropenem	31	2	22	81.8	35	3	17	94	37	1	17	98	37	1	17	98	37	0	18
Tobramycin	26	2	27	87	32	0	23	98	33	0	22	100	33	0	22	100	33	0	22
Levofloxacin	10	6	39	85.4	14	5	36	92.7	18	1	36	96.3	15	4	36	96.3	17	1	37

**Table 5:** Categorical agreement of direct AST (total no. of isolates=55) using breakpoints as per CLSI Reference Standard was either conventional antimicrobial susceptibility testing (AST) or VITEK following 16-18 hours of culture. Abbreviations: CLSI: Clinical and Laboratory Standards Institute; CA: categorical agreement; S: susceptible; I: intermediate; R: resistant. ^*^Interpretation using CLSI Breakpoints for direct AST. ^**^Interpretation using CLSI Breakpoints for culture based AST. ^a^Fosfomycin was analyzed only for *Escherichia coli.*

Anti-microbial agent	4 hours				6 hours				8 hours				16 hours				Reference Standard		
Interpretation as per CLSI	S	I	R	CA (%)	S	I	R	CA (%)	S	I	R	CA (%)	S	I	R	CA (%)	S	I	R
Ciprofloxacin^*^	-	-	-	-	-	-	-	-	14	3	38	81.8	14	3	38	81.8	7	3	45
Cotrimoxazole^*^	-	-	-	-	-	-	-	-	-	-	-	-	20	0	35	98.1	19	0	36
Piperacillin Tazobactam^*^	29		26	80	33	3	19	85.4	35	3	17	85.4	36	2	17	89	32	0	23
Cefoperazone Sulbactam^**^	11	15	29	58.1	25	9	21	83.6	34	1	20	94.5	33	2	20	100	33	2	20
Meropenem^*^	-	-	-	-	-	-	-	-	36	1	18	98	37	0	18	100	37	0	18
Fosfomycin^a**^	25	2	9	77.7	31	2	3	94.4	31	0	5	100	31	0	5	100	31	0	5
Cefepime^**^	3	16	35	58.1	13	10	32	78.1	21	4	30	92.7	21	4	30	92.7	25	1	29
Cefuroxime^**^	4	8	43	85.4	6	7	42	87.2	7	7	41	89	8	6	41	94.5	10	1	44
Netilmicin^**^	28	2	25	80	36	1	18	94.5	38	1	16	98.1	38	1	16	98.1	39	0	16
Tobramycin^*^	-	-	-	-	-	-	-	-	33	0	22	100	33	0	22	100	33	0	22
Ceftazidime^*^	-	-	-	-	-	-	-	-	15	8	32	83.6	18	5	32	87.2	23	3	29
Cefotaxime^*^	-	-	-	-	-	-	-	-	13	3	39	85.4	13	3	39	85.4	21	0	34
Nitrofurantoin^**^	29	6	20	70.9	36	4	15	83.6	42	2	11	92.7	42	3	10	96.3	44	1	10
Cefazolin^**^	11		44	98.1	12		43	100	12		43	100	12		43	100	12		43
Cefixime**	6	2	47	89	9	1	45	94.5	11	1	43	98.1	11	1	43	98.1	12	0	43

**Table 6:** Error rates and categorical agreement for Escherichia coli (total no. of isolates=36) using rapid antimicrobial susceptibility breakpoints as per EUCAST Reference standard was either conventional antimicrobial susceptibility testing (AST) or VITEK following 16-18 hours of culture. Abbreviations: RAST (EUCAST): Rapid Antimicrobial Susceptibility Testing (European Committee on Antimicrobial Susceptibility Testing); CA: categorical agreement; MnE: minor error; ME: major error; VME: very major error.

Anti-microbial agent	4 hours				6 hours				8 hours			
Interpretation as per RAST (EUCAST)	MnE	ME	VME	CA	MnE	ME	VME	CA	MnE	ME	VME	CA
Amikacin	8.3	0	0	91.6	5.5	0	0	94.4	5.5	0	0	94.4
Gentamicin	2.7	16	0	86.1	2.7	4	0	94.4	2.7	0	0	97.2
Tobramycin	5.5	15.3	0	83.3	0	3.8	0	97.2	0	0	0	100
Ciprofloxacin	2.7	33.3	10	86.1	8.3	0	13.3	80.5	8.3	0	13.3	80.5
Imipenem	11.1	16.6	0	75	11.1	3.3	0	86.1	2.7	0	0	97.2
Meropenem	2.7	20	16.6	77.7	2.7	3.3	0	97.2	2.7	0	0	97.2
Cotrimoxazole	0	35.7	0	10.3	0	14.2	4.5	91.6	0	0	4.5	97.2
Levofloxacin	5.5	30	0	86.1	5.5	0	0	94.4	5.5	0	0	94.4

**Table 7:** Error rates and categorical agreement for Escherichia coli (total no. of isolates=36) using breakpoints as per CLSI Reference standard was either conventional antimicrobial susceptibility testing (AST) or VITEK following 16-18 hours of culture. Abbreviations: CLSI: Clinical and Laboratory Standards Institute; CA: categorical agreement; MnE: minor error; ME: major error; VME: very major error. ^*^Interpretation using CLSI Breakpoints for direct AST. ^**^Interpretation using CLSI Breakpoints for culture-based AST.

Anti-microbial agent	4 hours				6 hours				8 hours				16 hours			
Interpretation as per CLSI	MnE	ME	VME	CA	MnE	ME	VME	CA	MnE	ME	VME	CA	MnE	ME	VME	CA
Netilmicin^**^	5.5	25	0	72	2.7	6.25	0	91.6	2.7	0	0	97.2	2.7	0	0	97.2
Tobramycin^*^	-	-	-	-	-	-	-	-	0	0	0	100	0	0	0	100
Ciprofloxacin^*^	-	-	-	-	-	-	-	-	11.1	0	10	80.5	11.1	0	10	80.5
Cefazolin^**^	0	12.5	0	97.2	0	0	0	100	0	0	0	100	0	0	0	100
Cefixime^**^	2.7	50	0	86.1	0	25	0	94.4	2.7	0	0	97.2	2.7	0	0	97.2
Cefepime^**^	36.1	28.5	0	47.2	19.4	14.3	0	72.2	8.3	4.7	0	88.8	8.3	4.7	0	88.8
Cefuroxime^**^	13.8	14.2	0	83.3	13.8	14.2	0	83.3	13.8	0	0	86.1	11.1	0	0	88.8
Ceftazidime^*^	-	-	-	-	-	-	-	-	11.1	10.5	0	83.3	8.3	5.2	0	88.8
Cefotaxime ^*^	-	-	-	-	-	-	-	-	8.3	29.4	0	77.7	8.3	29.4	0	77.7
Cefperazone-Sulbactam^**^	27.7	29.6	0	50	19.4	7.4	0	75	8.3	0	0	91.6	0	0	0	100
Meropenem^*^	-	-	-	-	-	-	-	-	2.7	0	0	97.2	0	0	0	100
Fosfomycin^**^	5.5	19.3	0	77.7	5.5	0	0	94.4	0	0	0	100	0	0	0	100
Nitrofurantoin^**^	8.3	17.6	0	75	8.3	5.8	0	86.1	2.7	0	0	97.2	0	0	0	100
Cotrimoxazole^*^	-	-	-	-	-	-	-	-	-	-	-	-	0	0	4.5	97.2

**Table 8:** Error rates and categorical agreement for Klebsiella pneumoniae (total no. of isolates=19) using rapid antimicrobial susceptibility breakpoints as per EUCAST Reference standard was either conventional antimicrobial susceptibility testing (AST) or VITEK following 16-18 hours of culture. Abbreviations: RAST (EUCAST): Rapid Antimicrobial Susceptibility Testing (European Committee on Antimicrobial Susceptibility Testing); CA: categorical agreement; MnE: minor error; ME: major error; VME: very major error.

Anti-microbial agent	4 hours				6 hours				8 hours			
Interpretation as per RAST (EUCAST)	MnE	ME	VME	CA	MnE	ME	VME	CA	MnE	ME	VME	CA
Amikacin	10.5	12.5	0	15.7	0	12.5	0	5.2	0	0	0	100
Gentamicin	0	22.2	0	89.4	0	0	0	100	0	0	0	100
Tobramycin	0	14.2	0	94.7	0	0	0	100	0	0	0	100
Ciprofloxacin	5.2	0	6.6	89.4	5.2	0	13.3	84.2	0	0	20	84.2
Imipenem	15.7	0	0	84.2	15.7	0	0	84.2	5.2	0	0	94.7
Meropenem	5.2	14.2	0	89.4	10.5	0	0	89.4	0	0	0	100
Cotrimoxazole	0	40	0	89.4	0	0	0	100	0	0	0	100
Levofloxacin	15.7	0	0	84.2	10.5	0	0	89.4	0	0	0	100

**Table 9:** Error rates and categorical agreement of Klebsiella pneumoniae (total no. of isolates=19) using breakpoints as per CLSI Reference Standard was either conventional antimicrobial susceptibility testing (AST) or VITEK following 16-18 hours of culture. Abbreviations: CLSI: Clinical and Laboratory Standards Institute; ​​​​​​CA: Categorical agreement; MnE: minor error; ME: major error; VME: very major error. ​^*^Interpretation using CLSI breakpoints for direct AST. ^**^Interpretation using CLSI breakpoints for culture-based AST.

Anti-microbial agent	4 hours				6 hours				8 hours				16 hours			
	MnE	ME	VME	CA	MnE	ME	VME	CA	MnE	ME	VME	CA	MnE	ME	VME	CA
Netilmicin^**^	0	14.2	0	94.7	0	0	0	100	0	0	0	100	0	0	0	100
Tobramycin^*^	-	-	-	-	-	-	-		0	0	0	100	0	0	0	100
Ciprofloxacin^*^	-	-	-	-	-	-	-		10.5	0	6.6	84.2	10.5	0	6.6	84.2
Cefazolin ^**^	0	0	0	100	0	0	0	100	0	0	0	100	0	0	0	100
Cefixime ^**^	5.2	0	0	94.7	5.2	0	0	94.7	0	0	0	100	0	0	0	100
Cefepime ^**^	21	0	0	78.9	10.5	0	0	89.4	0	0	0	100	0	0	0	100
Cefuroxime ^**^	10.5	0	0	89.4	5.2	0	0	94.7	5.2	0	0	94.7	5.2	0	0	94.7
Ceftazidime ^*^	-	-	-	-	-	-	-	-	5.2	0	0	94.7	5.2	0	0	94.7
Cefotaxime ^*^	-	-	-	-	-	-	-	-	0	0	0	100	0	0	0	100
Cefperazone-Sulbactam^**^	26.3	0	0	73.6	0	0	0	100	0	0	0	100	0	0	0	100
Meropenem^*^	-	-	-	-	-	-	-	-	0	0	0	100	0	0	0	100
Nitrofurantoin^**^	21	30	0	63.1	10.5	20	0	78.9	10.5	10	0	84.2	10.5	0	0	89.4
Cotrimoxazole^*^	-	-	-	-	-	-	-	-	-	-	-	-	0	0	0	100
